# Effects of Caffeine on Brown Adipose Tissue Thermogenesis and Metabolic Homeostasis: A Review

**DOI:** 10.3389/fnins.2021.621356

**Published:** 2021-02-04

**Authors:** Lachlan Van Schaik, Christine Kettle, Rodney Green, Helen R. Irving, Joseph A. Rathner

**Affiliations:** ^1^Department of Pharmacy and Biomedical Sciences, La Trobe Institute for Molecular Science, La Trobe University, Bendigo, VIC, Australia; ^2^Department of Physiology, School of Biomedical Sciences, The University of Melbourne, Melbourne, VIC, Australia

**Keywords:** caffeine, brown adipose tissue, thermogenesis, metabolic homeostasis, metabolsim

## Abstract

The impact of brown adipose tissue (BAT) metabolism on understanding energy balance in humans is a relatively new and exciting field of research. The pathogenesis of obesity can be largely explained by an imbalance between caloric intake and energy expenditure, but the underlying mechanisms are far more complex. Traditional non-selective sympathetic activators have been used to artificially elevate energy utilization, or suppress appetite, however undesirable side effects are apparent with the use of these pharmacological interventions. Understanding the role of BAT, in relation to human energy homeostasis has the potential to dramatically offset the energy imbalance associated with obesity. This review discusses paradoxical effects of caffeine on peripheral adenosine receptors and the possible role of adenosine in increasing metabolism is highlighted, with consideration to the potential of central rather than peripheral mechanisms for caffeine mediated BAT thermogenesis and energy expenditure. Research on the complex physiology of adipose tissue, the embryonic lineage and function of the different types of adipocytes is summarized. In addition, the effect of BAT on overall human metabolism and the extent of the associated increase in energy expenditure are discussed. The controversy surrounding the primary β-adrenoceptor involved in human BAT activation is examined, and suggestions as to the lack of translational findings from animal to human physiology and human *in vitro* to *in vivo* models are provided. This review compares and distinguishes human and rodent BAT effects, thus developing an understanding of human BAT thermogenesis to aid lifestyle interventions targeting obesity and metabolic syndrome. The focus of this review is on the effect of BAT thermogenesis on overall metabolism, and the potential therapeutic effects of caffeine in increasing metabolism via its effects on BAT.

## Introduction

Increases in the prevalence of overweight and obesity are a major health problem in people of all ages (Engin, 2017), with considerable concern in the rise of metabolic syndrome in children and adolescents (Gepstein and Weiss, 2019). Obesity is the accumulation of excess adipose tissue that has adverse impacts on physical healthy lifestyle patterns (Block et al., 2004; Drewnowski and Specter, 2004; Boardman et al., 2005; Abell et al., 2007; Gibson, 2011; Corral et al., 2012; Quick et al., 2013; Wang et al., 2013), and is positively correlated with poor health outcomes in cardiovascular disease, diabetes, cancer, and an array of musculoskeletal disorders (Anandacoomarasamy et al., 2008; Dehal et al., 2011; Liu et al., 2011; Willey et al., 2011; Ma et al., 2012; Vranian et al., 2012; Zhang and Rodriguez-Monguio, 2012). In addition to these chronic diseases, obesity is a major risk factor for higher risk of mortality due to COVID-19 (Pettit et al., 2020).

The development of obesity can largely be explained by an imbalance between caloric intake and total metabolic activity (Schwartz et al., 2017). Lifestyle interventions consisting of diet and exercise are common treatments for obesity, premised on the mistaken assumption that correcting the energy imbalance will lead to weight loss. However, there is a growing body of evidence suggesting that simple caloric restriction and exercise are insufficient on their own to promote and maintain weight loss (Greenway, 2015; Vettor et al., 2020). A lack of effective options for long-term weight reduction and subsequent weight maintenance exacerbates the enormity of the obesity problem; individuals who successfully complete behavioral and dietary weight-loss programs eventually regain most of the lost weight. A meta-analysis of 29 long term weight loss studies show that within 2 years more than half of the lost weight is regained, and by 5 years more than 80% of the lost weight is regained (Anderson et al., 2001). Consequently, identifying pharmacological therapies that may promote weight loss, through increased energy expenditure via thermogenesis, may be a way to augment current interventions for long term weight reduction (Ursino et al., 2009; Nedergaard and Cannon, 2010; Dulloo, 2011; Whittle et al., 2013).

Although the pathogenesis of obesity is far from fully understood, a large amount of effort is being undertaken to ameliorate obesity itself. Understanding the role of brown adipose tissue (BAT), in relation to human energy homeostasis and the potential to pharmacologically increase metabolism is an exciting and active research interest. Early estimates have suggested that increasing BAT thermogenesis in 50 g of BAT can increase metabolism by as much as 25% (Brooks et al., 2005). White adipose tissue (WAT) is distinctly different from BAT both in terms of physiology and embryonic development (Lee et al., 2013b). In simple terms, WAT stores energy, and BAT dissipates energy and releases heat (Cannon and Nedergaard, 2004). Surprisingly, in terms of development, BAT is closer in lineage to skeletal muscle rather than adipose tissue (Timmons et al., 2007; Cannon and Nedergaard, 2008; Seale et al., 2008; Kajimura et al., 2010; Lepper and Fan, 2010; Petrovic et al., 2010), with a large amount of mitochondria located in both BAT and skeletal muscle compared with WAT (Porter et al., 2016). This allows for the oxidative demand and heat production of activated BAT (Porter et al., 2016).

Activation of BAT thermogenesis is regulated by the sympathetic nervous system through β-adrenoreceptors in rodents, and is presumed to also be the case in humans (Cannon and Nedergaard, 2004). The neural pathway that controls the regulation of BAT thermogenesis has been thoroughly investigated and described in rats (Morrison and Nakamura, 2011). While there is overlap (Nazari et al., 2020), the neural pathway controlling the cardiovascular system appears to be different to that which controls thermoregulatory reflexes (Rathner et al., 2008). Sympathetic premotor neurons in the rostral raphe pallidus neurons regulate sympathetic outflow to BAT (Morrison, 2016) and are likely only weakly baroreceptor sensitive (Rathner et al., 2001), whereas the premotor neurons in the rostral ventrolateral medulla mediates baroreflex related sympathetic discharge (Morrison, 1999). Understanding the role of the different neural nuclei within the BAT regulatory pathway, particularly within the hypothalamus, is paramount to identifying pharmacological substances that may selectively activate BAT thermogenesis and increase energy expenditure, without necessarily altering whole body homeostasis.

Caffeine is the psycho-stimulant component of coffee, other beverages and various supplements (e.g., certain pre-workout powders) (Smith, 2002). There is ample evidence that caffeine increases thermogenesis acutely in both rodents and humans (Trimble, 1963; Velickovic et al., 2019), however the direct mechanism is unclear. Certain studies investigating the thermogenic and metabolic effects of caffeine have focused on caffeine acting directly on the BAT tissue at high doses (Astrup et al., 1990). However, these previous studies fail to consider that caffeine acts centrally to increase arousal (Ferre, 2010). This raises the possibility that previously reported caffeine evoked thermogenesis may have a central component, through acting on the thermoregulatory pathway underlying BAT thermogenesis. It remains uncertain if low, non-anxiogenic doses of caffeine, which activate arousal pathways centrally, are sufficient to activate thermogenesis. The focus of this review is on the effect of BAT thermogenesis on overall metabolism, and the potential therapeutic effects of caffeine in increasing metabolism via its effects on BAT.

## Therapeutic and Thermogenic Effects of Caffeine

Caffeine is a purine alkaloid (Ashihara et al., 2008) found in many food products and beverages and is likely the most widely consumed psycho-active drug worldwide being the psycho-stimulant component of coffee and other beverages and supplements (Smith, 2002). Caffeine has a number of physiological effects including increases in human endurance, physical performance, cognition, resting energy expenditure, and improvements in behavioral functions such as mood (Glade, 2010). Previous studies have demonstrated therapeutic effects of caffeine in metabolic parameters, hypertension, and hepatic fibrosis, which are components of metabolic syndrome. Caffeine (0.1% in drinking water) has been shown to decrease insulin concentrations and plasma glucose in rats fed high-fat and high-sucrose diets, respectively, with a decrease of mean arterial pressure in the two pathological models (Conde et al., 2012). Additionally, caffeine consumption attenuated weight gain adiposity in rats fed a high-fat diet (Conde et al., 2012). In human patients undergoing liver biopsy for clinical indications, a higher daily caffeine consumption is associated with a decrease in the severity of liver fibrosis (Modi et al., 2010).

Caffeine contained in coffee has been shown to induce lipolysis, thermogenesis, insulin secretion, and fat oxidation in both non-obese and obese humans (Astrup et al., 1990; Bracco et al., 1995; Acheson et al., 2004). In fact, consuming six cups of coffee (600 mg) within 12 h would be expected to induce a 100 kcal increase in daily energy expenditure (Dulloo et al., 1989). However, to date, there is little evidence that coffee consumption promotes significant weight loss in humans. This appears to be due to habituation to caffeine-induced catecholamine responses and lipolysis with prolonged use (Dekker et al., 2007).

Caffeine has been administered therapeutically in combination with ephedrine (mixed α and β-adrenoceptor agonist) to increase metabolism and activate BAT thermogenesis in rodents (Kim et al., 2011). Ephedrine itself has been shown to activate BAT thermogenesis in lean humans acutely but not in obese humans (Carey et al., 2013, 2015). However, both of these drugs have notable side effects, and caffeine at high doses is known to have significant adverse cardiovascular effects on heart rate and blood pressure (Hoehn-Saric and McLeod, 2000). Within the central nervous system, caffeine acts as an non-specific adenosine receptor antagonist (Ribeiro and Sebastiao, 2010). Previous research investigating caffeine’s physiological effects have generally focused on peripheral mechanisms (Astrup et al., 1990; Velickovic et al., 2019). However, in terms of arousal, caffeine’s effect is central (Ferre, 2010), suggesting that caffeine evoked thermogenesis may have a central component. In rodent studies, low (but stimulatory) doses of caffeine have previously activated orexinergic neurons in the dorsomedial hypothalamus and the perifornical areas of the lateral hypothalamus (Murphy et al., 2003; Sakurai, 2007). These regions of the hypothalamus have been shown to regulate sympathetic nerve activity to interscapular BAT, leading to thermogenesis in rodents (Tupone et al., 2011).

Caffeine works centrally to promote arousal through antagonism of the adenosine A_1__*A*_ receptor (Murphy et al., 2003), which releases orexinergic neurons from inhibition (Murphy et al., 2003). This suggests that central effects of caffeine on BAT thermogenesis are through its antagonistic action on the A_1__*A*_ receptor (Table 1). This observation is harmonious with a role of orexin/hypocretin as a key neuropeptide in the regulation of energy homeostasis and the sleep-wake cycle (Sakurai, 2007). It has been demonstrated that there is an increase in both brain adenosine and A_1__*A*_ receptor in the hypothalamus associated with the development of obesity in mice (Wu et al., 2017). Finally, antagonism of A_1__*A*_ receptor with systemic caffeine has been shown to reduce body weight, increase BAT thermogenesis and increase oxygen consumption in high fat diet-induced obese rats (Wu et al., 2017).

**TABLE 1:** Actions of adenosine receptor (AdR) subtypes (A_1A_, A_2A_, A_2B_, and A_3_) on thermogenesis.

**AdR Subtype**	**Principal Transduction**	**Central/Peripheral**	**Pre/Post Synaptic**	**Effect on Thermogenesis**
A_1A_	G_*i/*_G_*o*_ ↓ cAMP	Central	Pre	Central antagonism of the A_1A_ receptor increases BAT thermogenesis in rodents (Murphy et al., 2003).
		Peripheral	Post	Systemically lipolysis of brown adipocytes is inhibited after exposure to an A_1A_ receptor agonist in rodents (Woodward and Saggerson, 1986).
A_2A_	G_*s*_ family ↑ cAMP	Peripheral	Post	A_2A_ receptor agonists increase lipolysis and activate BAT thermogenesis in rodent and human brown adipocytes (Gnad et al., 2014). A_2A_ receptor agonists improve glucose tolerance, increase energy expenditure, and induce recruitment of brown adipocytes in rodents (Gnad et al., 2014).
A_2B_	G_*s*_ family ↑ cAMP	Peripheral	Post	A_2B_ receptor agonists increase lipolysis and activate BAT thermogenesis in rodent and human brown adipocytes (Gnad et al., 2014).
A_3_	G_*i/*_G_*o*_ ↓ cAMP	Peripheral	Post	Antagonism of A_3_ receptor has no significant effect on modulating lipolysis in rodent brown adipocytes (Gnad et al., 2014). A_3_ receptor KO mice have less abdominal and total body fat, and mice are protected from hypertension and cardiovascular diseases in a chronic kidney disease model (Yang et al., 2016).

The signaling of adenosine is transmitted via adenosine A_1_ and A_3_ receptors through G_*i*_/G_*o*_ family or by A_2__*A*_ and A_2__*B*_ receptors via G_*s*_ family G proteins (Table 1; Fredholm et al., 2011). The G_*i*__/_G_*o*_ family proteins inhibit adenylate cyclase (Francken et al., 1998) [which synthesizes cyclic adenosine monophosphate (cAMP) from ATP (Rivera-Oliver and Díaz-Ríos, 2014)], and G_*s*_ family stimulate adenylate cyclase (Graziano et al., 1987) thereby modulating the amount of cAMP available for downstream intracellular signaling. Activation of A_1_ or A_3_ receptors work through G_*i*__/_G_*o*_ family (Table 1) and lower cAMP whereas A_2__*A*_ or A_2__*B*_ work through G_*s*_ and increase cAMP (Cully, 2014). Lipolysis in WAT is inhibited via activation of A_1__*A*_ receptors (Johansson et al., 2008) which are abundantly expressed on WAT cells and act via G_*i*__/_G_*o*_ family to reduce cAMP (Trost and Schwabe, 1981; Mersmann et al., 1997; Tatsis-Kotsidis and Erlanger, 1999). Increases in intracellular cAMP promote lipolysis (Arner, 1976). Furthermore, adenosine A_2__*A*_ and A_2__*B*_ receptor agonists increase lipolysis and BAT thermogenesis (Gnad et al., 2014), whereas adenosine A_1__*A*_ receptor antagonism promotes increases in heart rate and oxygen consumption. Expression of A_2__*A*_ receptors is increased in cold-exposed mice as well as in brown adipocytes in response to norepinephrine or intracellular cAMP (Gnad et al., 2014). As caffeine is a non-specific adenosine receptor antagonist it may influence multiple peripheral mechanisms that act paradoxically upon adipose tissue and thermogenesis. As such there remains potential to investigate effects of combination therapy of caffeine and selective adenosine A_2__*A*_ or A_2__*B*_ agonists on thermogenesis. Such a combination therapy may increase the efficacy of caffeine.

In addition to antagonizing adenosine receptors, caffeine is a non-selective phosphodiesterase inhibitor (Moustafa and Feldman, 2014). Phosphodiesterase converts cAMP to adenosine monophosphate, thus caffeine’s action on phosphodiesterase increases intracellular cAMP (Boswell-Smith et al., 2006). Raising cellular cAMP could possibly be a peripheral mechanism by which caffeine increases UCP1 (uncoupling protein 1, which facilitates a futile cycle in the mitochondria of brown/beige adipocytes) activity (Fukano et al., 2016). β3-adrenoceptors are G protein coupled receptors linked to G_*s*_ proteins. Activation of β3-adrenoceptors leads to increased cAMP production, which in turn activates protein kinase A, which stimulates lipolysis, releasing free fatty acids and activating UCP1 (Himms-Hagen, 1989; Cannon and Nedergaard, 2004). Additional experiments investigating the effects of protein kinase A inhibition or cAMP concentration may provide further information on the role of cAMP levels in the caffeine response. As such there remains potential to combine stimulatory doses of caffeine with β3-adrenoceptor agonists.

Intake of caffeine improves fructose-induced insulin resistance and hypertension by enhancing central insulin signaling in rats (Yeh et al., 2014). This is through enhancing insulin sensitivity within the nucleus of the solitary tract to prevent hypertension by increasing nitric oxide production. Adenosine A_2__*A*_ receptor signaling pathway in the nucleus of the solitary tract mediates nitric oxide production related with the control of blood pressure (Ho et al., 2008). This is important because the nucleus of the solitary tract has received considerable attention for its role in the regulation of metabolism and body temperature (Székely, 2000; Cao et al., 2010; Grill and Hayes, 2012). Activation of the adenosine A_1__*A*_ receptor within the neurons of the nucleus of the solitary tract leads to a hypometabolic state, inhibiting BAT thermogenesis, reducing energy expenditure and inhibiting shivering thermogenesis in rats (Tupone et al., 2013). Suggesting that chronic activation of adenosine A_1__*A*_ receptors (lowering cAMP levels) in the nucleus of the solitary tract can reduce energy expenditure and induce a metabolic imbalance potentially pre-disposing individuals to obesity and metabolic disease. As caffeine is an antagonist at the adenosine A_1__*A*_ receptor, perhaps these effects may be attenuated or reversed with caffeine treatment. Interestingly, chronic caffeine consumption diminishes diabetic symptoms by increasing insulin sensitivity via more efficient insulin signaling (Park et al., 2007). Moreover, caffeine alleviates fructose-induced metabolic disturbances in rats (Yeh et al., 2014). However, several researchers have concluded that caffeine consumption may increase blood pressure, thus leading to an elevated risk of hypertension (Savoca et al., 2004; Noordzij et al., 2005). Conversely, most epidemiological studies have shown that the habitual intake of caffeinated coffee does not increase the risk of hypertension, (Savoca et al., 2004; Geleijnse, 2008) and chronic caffeine intake has been found to prevent diet-induced hypertension in rats (Conde et al., 2012). These observations suggest a central mechanism for caffeine evoked BAT thermogenesis and increases in energy expenditure, that have possible applications in the clinical care of individuals with metabolic disease, hypertension, and other obesity related diseases.

## Physiology of Adenosine Receptors – As a Mechanistic Understanding of the Therapeutic Effects of Caffeine

The purinergic nucleoside signaling molecule adenosine is found within the sympathetic nervous system (Gourine et al., 2009) and in the central nervous system, and is both a precursor and breakdown product of adenosine-triphosphate (Abbracchio et al., 2009). The A_2__*A*_ receptor is highly expressed on BAT and appears to have a major role in BAT activation and recruitment of brown adipocytes (Gnad et al., 2014) which involves the proliferation and differentiation of precursor cells, and also hypertrophy of mature brown adipocytes. A_2__*A*_ receptor agonists increase lipolysis in both murine and human brown adipocytes (Gnad et al., 2014) as downstream G_*s*_ family G proteins will be activated raising cellular cAMP. Additionally, these same A_2__*A*_ receptor agonists improved glucose tolerance, increased energy expenditure, increased [^18^F]fluorodeoxyglucose (FDG) uptake in BAT, induced recruitment of brown adipocytes and protected mice from diet-induced obesity (Gnad et al., 2014). These findings suggest promising thermogenic effects of adenosine via direct action on BAT. Previous studies of the effects of adenosine on brown adipocytes show lipolysis is inhibited in rodents after exposure to an A_1__*A*_ adenosine receptor agonist (Woodward and Saggerson, 1986; Table 1).

## The Complex Physiology of Adipose Tissue

“Adipose tissues” found in mammals contain three types of adipocytes (white, brown, and beige/brite) and are categorized into two types of tissue (WAT and BAT) (Lee et al., 2013b; Wu and Spiegelman, 2013). White adipocytes predominantly make up WAT, with a smaller number of beige adipocytes mixed in Lee et al. (2013b), and BAT being predominantly brown adipocytes (Lidell et al., 2013; Rosenwald and Wolfrum, 2014). Some tissue plasticity occurs because in humans these brown adipocytes seem to be displaced by white adipocytes under conditions of aging (Contreras et al., 2014; Zoico et al., 2019). However, it is unclear whether these are truly white adipocytes or if there is some form of reverse browning. While historically WAT was considered simply as a store of energy (in the form of triglycerides), it is now known that it can also act as an endocrine organ and secrete adipokines (Coelho et al., 2013). Adipokines are pro-inflammatory chemical signaling molecules secreted by adipose tissue (Mancuso, 2016). In obese patients, these adipokines contribute to low-grade systemic inflammation (Tilg and Moschen, 2006). Progranulin, lipocalin−2, adiponectin, and leptin are adipokines which link obesity to the immune system, and are potential therapeutic targets in obesity-related diseases, such as diabetes mellitus (Reinehr and Roth, 2018), rheumatoid arthritis (Carrion et al., 2019), and osteoarthritis (Tu et al., 2019).

Each class of adipocyte is functionally, biochemically, and morphologically distinct. Beige adipocytes are typically dispersed in WAT (Zoico et al., 2019) and, inactivated, can look indistinguishable from their white adipocyte neighbors. However, sympathetic activity, can induce these cells to “brown” (Young et al., 1984; Sidossis et al., 2015), which is associated with increased whole-body metabolic rate (Sidossis et al., 2015), and this browning of WAT is attenuated in β3-adrenoceptor knockout mice (Jimenez et al., 2003). Browning is a term that describes the emergence of beige adipocytes in WAT and is a reversible process which represents adaptation to increased thermogenic demand. Morphological differences are easily observed between BAT and WAT, but are less obvious between white adipocytes and beige adipocytes (Ikeda et al., 2018). White adipocytes have a large single lipid droplet, containing energy stored as triglycerides, with the nucleus displaced to the periphery of the cell (Cinti, 2006). Similar to white adipocytes, beige adipose tissue are unilocular, however have smaller and multiple lipid droplets (Park et al., 2014). In contrast, brown adipocytes typically have a polygonal shape, and multilocular lipid droplets (de Jong et al., 2019).

The embryonic development of adipocytes, both white and brown, is common with skeletal muscle, other connective tissues, and bone that are all generally accepted to arise from progenitor cells of the mesoderm (Gesta et al., 2007; Billon et al., 2008). Several studies indicate that the formation of BAT shares a closer relationship with skeletal muscle, rather than WAT (Timmons et al., 2007; Cannon and Nedergaard, 2008; Seale et al., 2008; Kajimura et al., 2010; Lepper and Fan, 2010; Petrovic et al., 2010). White adipocytes derive from lateral mesoderm, and are myf-5 negative precursor cells (Sanchez-Gurmaches and Guertin, 2014). This contrasts with brown adipocytes which derive from myf-5 positive precursor cells of the paraxial mesoderm (Sanchez-Gurmaches and Guertin, 2014; Ikeda et al., 2018). Myf-5 is a necessary gene transcription regulator for the development of myoblast (Ustanina et al., 2007). Cold exposure is known to induce proliferation of rodent vascular endothelial cells and pre-adipocytes (Bukowiecki et al., 1986; Geloen et al., 1992; Klingenspor, 2003; Lee et al., 2015) in BAT. The pre-adipocytes differentiate into mature brown adipocytes, resulting in BAT hyperplasia and enhanced BAT function. It is interesting to speculate that in humans, in our centrally heated houses we may keep newborns too warm, perhaps providing an environmental setting reducing brown adipocyte proliferation contributing to the emergence of obesity and type II diabetes in adolescents. Consistent with this, rat pups reared for 8 weeks in cool environment (18°C) and then housed in a thermoneutral environment for 4 weeks had 15% greater interscapular BAT mass than rats reared in 30°C environment prior to housing at thermoneutrality (Morrison et al., 2000). Additionally, rodent mature brown adipocytes have the ability to proliferate upon the activation of β3-adrenoceptor, as increases in the number of brown adipocytes expressing UCP1 following cold exposure (Fukano et al., 2016). This is confirmed as inhibition of β3-adrenoceptor with an antagonist (SR59230A, 1 mg/kg) reduces the number of proliferating brown adipocytes during cold exposure of 10°C (Fukano et al., 2016).

Both rodent and human brown adipocytes contain large numbers of mitochondria (Porter et al., 2016), and therefore skeletal muscle and BAT have similar oxidative capacities (Porter et al., 2016). Furthermore, compared to WAT, BAT is also highly vascularized in humans, this allows for the oxidative demand and heat dissipation into the body (Cypess et al., 2009).

## Effect of Bat Thermogenesis on Metabolism

The finding that BAT activity (Vijgen et al., 2011; Madden and Morrison, 2016; Loh et al., 2017) and volume (Leitner et al., 2017) is inversely related with adiposity in adult humans (Wang et al., 2015) has encouraged the assessment of the role of BAT in metabolic regulation. There is evidence that the metabolic activity of BAT can offset some of the energy imbalance associated with weight gain. Estimates of energy consumption are made on the basis of some experimentally derived assumptions (Carpentier et al., 2018), as indicated in Table 2, suggest that BAT thermogenesis can contribute up to 10% of resting energy expenditure. This is consistent with measured increases in energy expenditure in humans after β3-adrenoceptor activation (Cypess et al., 2015; O’Mara et al., 2020). Interestingly, comparisons between summer and winter seasons in adult humans following mild cold exposure (15°C) for 3 h, shows an increase in non-shivering thermogenesis and resting metabolic rate by 7% in summer and 11.5% in winter (van Ooijen et al., 2004). While increasing non-shivering thermogenesis does not exactly mean increasing BAT thermogenesis, it is certainly a component. The higher metabolic response in winter compared with summer indicates cold acclimatization, that is, adaption of non-shivering thermogenesis over time. This suggests that acute studies on BAT may not be sensitive enough to detect a maximal BAT response as environmental conditions may impact on the total measured energy consumption. Studies investigating a thermogenic response following chronic exposure to a treatment may yield compounded results over time.

**TABLE 2:** Brown adipose tissue (BAT) oxidative metabolism and contribution to total body energy expenditure.

	**Room temperature (22°C)**			**Mild cold exposure (**15**–18°C)**		
Oxidative metabolism per g of tissue	0.007 ml/g/min			0.012 ml/g/min		
Lower BAT mass	3.3 ml/min			5.6 ml/min		
Higher BAT mass	15.3 ml/min			26.8 ml/min		
Energy Expenditure at 4.8 kcal/L of O_2_ consumed	Room temperature (22°C)			Mild cold exposure (15–18°C)		
Lower BAT mass	0.0158 kcal/min	22.7 kcal/day	1% REE	0.027 kcal/min	38.9 kcal/day	2% REE
Higher BAT mass	0.073 kcal/min	105.1 kcal/day	6% REE	0.125 kcal/min	180 kcal/day	10% REE

*Resting energy expenditure (REE) assumes thermoneutrality which means zero (0) BAT activity. Calculations assume an energy expenditure of 4.8 kcal per L of oxygen consumed (Leonard, 2010) and an adipose tissue density of 0.925 g/mL (Martin et al., 1994). Values are derived from the literature for BAT oxygen consumptions (u Din et al., 2016), BAT mass range (Leitner et al., 2017), and Resting Energy Expenditure (REE = 1.2 kcal/min = 1728 kcal/day) is based on a 70 kg male (Brooks et al., 2005).*

Clear evidence of the mechanism for BAT activation in humans remains to be determined. Increases in the thermogenic capacity of humans and rodents can occur in response to repeated or long term exposure to cold stimuli or pharmacological activation with β3-adrenergic receptor agonists *in vivo* (Cousin et al., 1992; Yoneshiro et al., 2013; Cypess et al., 2015; Hanssen et al., 2016; Finlin et al., 2018; O’Mara et al., 2020). Part of the increased thermogenic capacity is due to the browning of WAT (Contreras et al., 2016). Therefore, prolonging the activation of BAT can increase BAT mass (Cannon and Nedergaard, 2004), resulting from proliferation and hypertrophy of beige adipocytes which trigger browning of WAT (Wankhade et al., 2016).

Obesity results in increased storage of triglycerides in both skeletal muscle cells (Goodpaster et al., 2000) and brown adipocytes (Shimizu et al., 2014). The increased storage of triglycerides has negative effects on lipolysis and inhibits glucose metabolism, leading to reduced muscle (and BAT) glucose utilization (Boden et al., 2001; Boden, 2003, 2006). Activation of BAT utilizes free fatty acids as a substrate (Penicaud et al., 2000), thus increasing fat catabolism. Prolonged BAT activation in humans improves insulin sensitivity (Finlin et al., 2018), glucose metabolism and insulin secretion (O’Mara et al., 2020). Increases in circulating plasma free fatty acids results in impaired insulin secretion (Kashyap et al., 2003), and insulin resistance (Boden et al., 2001; Boden, 2006), thereby increasing blood glucose levels. Therefore, activation of BAT and beige adipocytes have the potential to address the lipid and glucose imbalance associated with metabolic disorders in humans such as type 2 diabetes mellitus with significant implications for health care.

## The Primary β-Adrenoreceptor Involved in Human Bat Activation (β1, β2, or β3)

Sympathetic nervous system activity, via β-adrenergic receptors is a significant regulator of BAT thermogenesis (Cannon and Nedergaard, 2004). The primary β-adrenoreceptor involved in human BAT activation remains contentious. In rodents, BAT is primarily induced through β3-adrenoreceptor stimulation (Bachman et al., 2002) although β1-adrenoceptors can provide a functionally compensatory mechanism in β3-adrenoceptor knockout mice (Chernogubova et al., 2005). β3-adrenoceptors were also thought to be the primary adrenoceptor of human brown adipocytes (Cypess et al., 2015). *In vivo* treatment with the β3-agonist mirabegron [approved for human use in the treatment of overactive bladder (Khullar et al., 2013)] at a dose of 200 mg, four times the recommend therapeutic dose, increases whole body energy expenditure and BAT thermogenesis as detected by [^18^F] fluorodeoxyglucose (^18^ FDG, glucose PET analog) uptake (Cypess et al., 2015; Baskin et al., 2018; O’Mara et al., 2020). However, these doses are accompanied by significant increases in heart rate and mean arterial pressure (Cypess et al., 2015; Baskin et al., 2018; Finlin et al., 2018; O’Mara et al., 2020), possibly implying that at such doses mirabegron is not selective to β3-adrenoceptors. Mirabegron has 446 times higher affinity for β3 than β1 or β2-adrenoceptor subtype (Takasu et al., 2007).

Since the β3-adrenoreceptor is highly expressed in few organs, such as the urinary bladder, gall bladder, and BAT and, significantly, is absent from blood vessels, specifically targeting the β3-adrenoreceptor may be an attractive option for therapeutically augmenting non-shivering thermogenesis, as it would be expected that there may be limited adverse effects (Schena and Caplan, 2019). Evidence for a physiological effect of β3-adrenergic receptor activity on the human heart is still being debated (Berkowitz et al., 1995; Gauthier et al., 1996, 1999; Schena and Caplan, 2019). β3 adrenergic receptors decrease the force of contraction of the ventricles (Tavernier et al., 2003) and promote release of nitric oxide (Calvert et al., 2011), which in turn may have positive impact on cardiac health.

Other β3-adrenoceptor agonists that show effects in rodents, have limited effects when tested on humans (Weyer et al., 2008). Various β3-adrenoreceptor agonists, such as L-796568 (Larsen et al., 2002), ZD7114 and ZD2079 (Buemann et al., 2000), Ro 40-2148 (Jequier et al., 1992), and BRL 26830 (Connacher et al., 1988, 1992) have been used in clinical trials to test for efficacy to induce weight loss, but have produced limited effects. Furthermore, unforeseen effects such as tremor, and tachycardia were observed (Connacher et al., 1990), suggestive of potential β1- and β2-adrenoceptor co-activation. The β3-adrenoceptor is expressed in both white and brown adipocytes in both rodents and humans (Chamberlain et al., 1999; Schena and Caplan, 2019), however in humans β3-adrenoceptor mRNA levels are much lower (Schena and Caplan, 2019; Blondin et al., 2020). While *in vitro* analysis of brown adipocytes reveals that β1-adrenoreceptors are the predominant adrenoreceptor in both an immortalized cell line and human BAT biopsies (Riis-Vestergaard et al., 2020). Furthermore, immunohistochemical analysis identifies β3-adrenoceptors in intact human adipocytes and ventricular myocardium, consistent with evidence that β3-adrenoceptors can mediate lipolysis in human white adipocytes (De Matteis et al., 2002), and a negative inotropic effect within the ventricular myocardium (Gauthier et al., 2000). Thus, in contrast to rodent BAT the adrenergic receptors involved in humans are still being debated. These differences between species may be due to multiple factors, such as differences in the ligand-binding pocket in the β3-adrenoceptor, expression patterns of β3-adrenoceptor or cross-reactivity with the β1-adrenoceptor causing cardiovascular adverse effects in humans, or bioavailability (Arch, 2011; Dehvari et al., 2018; Loh et al., 2019).

In humans, BAT activation can be inhibited with treatment of the β-adrenergic antagonist propranolol (80 mg), at doses typically used to inhibit β1-adrenoceptors (Soderlund et al., 2007). Propranolol is a significantly weaker antagonist of β3- than β1- or β2-adrenoceptors (Baker, 2005). The implication of this, is that human BAT thermogenesis may be mediated through β1- or β2-adrenoceptors, even though β3-adrenoceptors are present. However, whether the doses of propranolol used were high enough to block not only β1- but also β3-adrenoceptors or if human BAT is stimulated mainly via β1-adrenoceptors, is not clarified in this study (Soderlund et al., 2007). Propranolol has higher selectivity for β2- rather than β1-adrenoceptors (Baker, 2005), raising the possibility that it is the β2- not β1-adrenoceptor largely involved in this response. These questions can largely be addressed, as there are specific β1-adrenoceptor antagonists, such as CGP 20712A, which has ∼1,000–10,000-fold selectivity for the β1-adrenoceptor (pK_*B*_, ∼8.4–9.5) relative to the β2- (pK_*B*_ ∼5.1) and β3-adrenoceptors (pK_*B*_ ∼4.2–4.7) (Seifert, 2011).

*In vitro* analysis of brown adipocytes shows that UCP1 mRNA expression is raised 6 to 12 fold by dobutamine, a β1-adrenoceptor agonist, and eight fold by isoproterenol (a non-selective β-adrenoceptor agonist), whereas neither procaterol (β2-adrenoceptor agonist), CL314.432, or mirabegron (β3-adrenoceptor agonists) affected UCP1 (Riis-Vestergaard et al., 2020). Isoproterenol induced UCP1 mRNA expression is attenuated by 62.5%, whereas isoproterenol induced UCP1 mRNA levels were unaffected by siRNA silencing the β3-adrenoceptor (Riis-Vestergaard et al., 2020). Together these results suggest that adrenergic stimulation of UCP1 may mainly be mediated through β1-adrenoreceptors (Riis-Vestergaard et al., 2020), however, the role of β2-adrenoceptor was not assessed and requires clarification.

Formoterol is a highly subtype selective β2-adrenoceptor agonist with 646 times higher affinity for human β2 over β3, and 331 times higher affinity for β2 over β1-adrenoceptor (Baker, 2010). Brown adipocyte oxygen consumption was increased significantly by formoterol and knock down of the β2-adrenergic receptor impaired thermogenesis (Blondin et al., 2020). Formoterol promoted BAT thermogenesis *in vitro*, that could not be achieved using therapeutic doses of the β3 agonist mirabegron (50 mg), but was at 200 mg (Blondin et al., 2020). Increases in energy expenditure, fatty acid oxidation stimulation and thermogenesis in response to oral administration of formoterol (160 μg) (Lee et al., 2013a), are similar to what is observed with mirabegron (200 mg) (Blondin et al., 2020). However, the source of thermogenesis was not determined following administration of formoterol. Although, oral administration of formoterol increases energy expenditure by 13% and fat utilization by 23%, without inducing tachycardia, six out of eight participants reported palpitation, tremor, two lost appetite, and one experienced insomnia (Lee et al., 2013a).

While the findings from Blondin et al. (2020) provide evidence for β2-adrenergic stimulation of brown adipocytes *in vitro*, there is still a role for the other adrenoceptors. Chronic mirabegron [100 mg being the dose for maximal efficacy (Chapple et al., 2013)], treatment for 4 weeks increased BAT activity in healthy non-obese females (O’Mara et al., 2020). The initial dose of mirabegron on day 1 increased participants resting energy expenditure by 10.7%; interestingly the baseline resting energy expenditure on day 28 was 5.8% higher than the baseline resting energy expenditure prior to drug exposure (O’Mara et al., 2020). However, participants in this study served as their own control and showed acute increases in heart rate (6 beats per minute) and systolic blood pressure (8 mmHg) (O’Mara et al., 2020). Though, it should be clearly stated that these acute cardiovascular effects are not apparent following chronic (28 day) treatment of the drug (O’Mara et al., 2020). Similarly increases in systolic blood pressure of 10 mmHg and heart rate of 11 beats per minute, were observed following acute administration of mirabegron (200 mg) (Blondin et al., 2020). Although these increases in systolic blood pressure and heart rate are not seemingly large, a 10 mmHg increase in systolic blood pressure to a hypertensive patient may in fact be substantial and will likely reduce the use of mirabegron (100 mg) clinically in obese patients. As such, perhaps a future experiment administering mirabegron to patients on β1- and β2-adrenoceptor antagonists may ameliorate the previously reported cardiovascular effects. There is also potential for a more selective β3-agonist to improve these apparent adverse cardiovascular effects as mirabegron exerts a cardio-stimulant and cardio-depressant effect which is unrelated to β3-adrenoceptor activation (Mo et al., 2017). Additionally, a combination therapy of a β3-agonist with stimulatory, but non-anxiogenic doses of caffeine remains a possibility.

Nonetheless, it is possible human BAT express all three β-adrenoceptors subtypes or there is a bias in how a β-adrenoceptor behaves that influences its response. Surprisingly, there has been little work undertaken investigating the primary β-adrenoreceptor in human BAT. This is potentially due to a limited access of BAT biopsy material for both primary cell cultures and explant cultures (Lee et al., 2016). Further investigations using different β-adrenoceptor agonists *in vivo* or a combination of agonists and antagonists are needed to understand receptor composition of β-adrenoceptors in human BAT. There is much to be learned regarding pharmacological BAT activating agents that mimic the effects of cold exposure in humans, as whole body and BAT thermogenesis can both increase in response to cold stimuli, while heart rate decreases significantly (Cypess et al., 2012). In addition, increases in BAT glucose uptake and whole-body energy expenditure can occur independently of direct stimulation of BAT thermogenesis (Blondin et al., 2017).

## Sympathetic Control of Bat

The regulation of sympathetic activity provides a complex homeostatic mechanism that precisely regulates the functional crosstalk of organs involved in balancing energy expenditure and caloric intake (Villarroya and Vidal-Puig, 2013). In hamsters, sympathetic nerve activity to WAT drives lipolysis, this provides free fatty acids for sympathetically mediated increases in BAT thermogenesis (Brito et al., 2008), suggesting that increasing BAT activity will have direct lipolytic consequences.

The neural mechanisms involved in the thermoregulatory control of BAT are well understood in rodents (Morrison, 2016). There is circumstantial evidence that human BAT is under sympathetic control, as human brown adipocytes express adrenoreceptors (Virtanen et al., 2009; Cypess et al., 2015; Blondin et al., 2020), certain adrenergic agonists have led to BAT thermogenesis (Kim et al., 2011; Cypess et al., 2015) and recent evidence of a nerve supply to BAT-like tissue in humans (Sievers et al., 2020). However, there is a lack of understanding of the physiological signals that drive sympathetic nerve activity to BAT in humans. Evidence suggests that mild environmental cooling (14–19°C) (Cypess et al., 2012; Chen et al., 2013) is adequate to activate BAT thermogenesis.

Based on the limited data available from neuroimaging studies the neural circuit underlying thermoregulation in humans, seems to involve the cerebral cortex and hypothalamus (Egan et al., 2005). Additionally, human rostral medullary raphé neurons are selectively activated in response to a thermoregulatory challenge and point to the location of thermoregulatory neurons similar to those of the raphé pallidus nucleus in rodents (McAllen et al., 2006). A human cadaveric study has also identified a nerve branch to supraclavicular tissue with a similar morphology to BAT, histological analysis of the tissue shows tyrosine hydroxylase immunoreactive structures, which likely represent sympathetic axons (Sievers et al., 2020). These points, taken together, suggest that certain structures that are involved in rodent neural circuitry underlying thermoregulation are also involved in the human circuitry. However, additional research is needed to draw comparisons between the two.

Orexins (hypocretins) are neuropeptides that are synthesized in specific neurons located in the lateral hypothalamus, and perifornical lateral hypothalamus. Activation of the lateral hypothalamic neurons significantly increases BAT sympathetic nerve activity in rodents (Cerri and Morrison, 2005). Orexinergic neurons in the perifornical lateral hypothalamus project to the rostral raphe’ pallidus. This increases the excitability of BAT sympathetic premotor neurons. Orexins have a recognized role in the management of body temperature and controlling heart rate, energy expenditure and BAT thermogenesis (Cao and Morrison, 2003; Sellayah et al., 2011; Girault et al., 2012; Messina et al., 2014). Additionally, direct injection of orexin into the rostral raphe’ pallidus increases BAT sympathetic nerve activity in rats (Madden et al., 2012). In rat studies, low (but stimulatory) doses of caffeine have previously activated orexinergic neurons in the dorsomedial hypothalamus and the perifornical areas of the lateral hypothalamus (Murphy et al., 2003; Sakurai, 2007), suggesting a possible central mechanism for caffeine evoked thermogenesis.

Recently the model for sympathetic control of BAT in rodents proposed by Morrison (2016) has been challenged (Saper and Machado, 2020). A series of studies in mice have demonstrated that pre-optic neurons that trigger cooling when activated, express a number of genetic markers, involving several that encode a protein fragment named pyroglutamylated RF-amide peptide (Wang et al., 2019; Hrvatin et al., 2020; Takahashi et al., 2020). Pyroglutamylated RF-amide peptide is a hypothalamic neuropeptide also expressed in median pre-optic neurons (Takahashi et al., 2020). The suggestion is that key neurons involved in causing hypothermia are not located in the medial pre-optic area, but are potentially found in the median pre-optic area and project directly to the dorsomedial hypothalamus. However, the data from the Morrison model is primarily from rat studies (Morrison et al., 2014). It is entirely possible that the neural organization in mice is different to that in rats. For example, in rats cutaneous vasoconstriction is not dependent on the dorsomedial hypothalamus in response to cold stimuli or activation of the febrile response (Rathner et al., 2008). A finding that is ignored by the proposed new model and has not been replicated in mice. A recent study demonstrates that inhibition of the median pre-optic area prevents cold evoked BAT thermogenesis in rats suggesting that a population of neurons within the median pre-optic area are required to be activated to drive BAT thermogenesis (da Conceição et al., 2020). This identifies the median pre-optic area as a primary source of glutamatergic excitation of BAT sympatho-excitatory neurons within the dorsomedial hypothalamus (da Conceição et al., 2020). It is yet to be determined whether these hypothalamic projecting neurons within the median pre-optic area excite thermogenesis promoting dorsomedial hypothalamic neurons during prostaglandin E_2_ or caffeine evoked BAT thermogenesis. Caffeine may act on neurons within the median pre-optic area, as this nuclei involves both sleep-wake neurons (Vanini et al., 2020) and energy metabolism neurons (da Conceição et al., 2020).

The protein fragment pyroglutamylated RF-amide is known to activate the G protein-coupled receptor, GPR103, which mediates orexigenic effects in rodents (Chartrel et al., 2003), and has previously been implicated in food intake, sympathetic regulation and anxiety (Takayasu et al., 2006; Okamoto et al., 2016). Central administration of pyroglutamylated RF-amide stimulates high fat food intake in rodents (Primeaux et al., 2008). In addition to stimulating food intake, pyroglutamylated RF-amide reduces thermogenesis, and increases body weight, fat mass and adipogenesis (Moriya et al., 2006; Mulumba et al., 2010). Given the role of pyroglutamylated RF- amide in orexin signaling and anxiety, there remains a possibility that caffeine may influence the effect of this protein. The proposed new model of thermoregulation, involving a population of excitatory neurons in the median pre-optic area expressing pyroglutamylated RF-amide, that connect directly to the dorsomedial hypothalamus (Hrvatin et al., 2020; Takahashi et al., 2020), is largely based on genetic molecular observations in knockout mouse models. Although knockout models can create odd effects as systems balance to cope with a lack of gene product (Teng et al., 2013), these observations are further validated using pharmacological and electrophysiological procedures on rats (da Conceição et al., 2020). Together, these findings provide new insights into the complexity of sympathetic thermoregulatory circuit. Gaining a better understanding of this central circuit is particularly important in developing new therapeutic approaches for augmenting BAT thermogenic energy expenditure, to improve energy homeostasis.

Interestingly, control of BAT in rats appears to follow a neural pathway which is exclusive and distinct from that which controls the cardiovascular system (Morrison, 1999). Additionally, there is evidence that indicates the premotor neurons in the central nervous system that regulate BAT are in fact specific and are separate from those regulating cutaneous vasomotor activity (Rathner et al., 2008). Studies that have observed increased interscapular BAT activity in rodents through increased nerve activity have demonstrated that this increase is accompanied by increases in heart rate and mean arterial pressure (Cerri and Morrison, 2005; Tupone et al., 2011). However, central, and systemic administration of stimulatory, but non-anxiogenic doses of caffeine increases interscapular BAT temperature in rats, without increasing core temperature, or increasing heart rate and mean arterial pressure (Van Schaik et al., 2021). These findings are further strengthened by increased neuronal activity, as measured by c-Fos-immunoreactivity within subregions of the hypothalamic area, previously implicated in regulating BAT thermogenesis. These include the perifornical area of the lateral hypothalamus, the lateral hypothalamus, and the dorsomedial hypothalamus following doses of caffeine administered either systemically or centrally (Van Schaik et al., 2021). These are areas known to that contain orexinergic neurons (Murphy et al., 2003; Berthoud et al., 2005; Cerri and Morrison, 2005; Tupone et al., 2011). The direct effects of caffeine in each of these brain regions remain unclear, and whether doses of caffeine comparable to the doses used in this study can activate BAT thermogenesis, without a cardio-dynamic effect in humans is unclear. However, it is clear that caffeine, at stimulatory doses act via the central nervous system to increase BAT thermogenesis in rodents.

This suggests that non-selective sympathetic activators may be beneficial as weight control agents. However, there remains a certain amount of risk with global sympathetic activation. Historically, therapeutic drugs that have been successful in creating negative energy balance weight loss in humans via non-selective activation of the sympathetic nervous system have been associated with cardiovascular side effects (Table 3). These adverse effects have prevented their use clinically (Yen and Ewald, 2012). For future sympathetic based strategies to increase energy expenditure through activation of BAT to be clinically successful, they will need to firstly enhance the sensitivity of BAT to the sympathetic nervous system or selectively stimulate BAT sympathetic nerve activity. These strategies may ameliorate the negative cardiovascular effects of non-selective sympathetic activation, while offering new ways for specifically promoting energy expenditure and decreasing the metabolic compensatory responses to chronic caloric restriction.

**TABLE 3:** Weight loss agents via non-selective activation of the sympathetic nervous system.

**Agents**	**Mechanism of action**	**Side effects**
Fenfluramine/Phenteramine (FenPhen)	Inhibit serotonin reuptake Promote serotonin release Sympathomimetic agonist	Valvular disease (Connolly et al., 1997; Teramae et al., 2000). Primary pulmonary hypertension (Abenhaim et al., 1996).
Sibutramine	Norepinephrine and serotonin reuptake inhibitor	Mania/panic attacks/psychosis (Perrio et al., 2007). Myocardial infarction (James et al., 2010).
Ma Huang/Ephedra	Sympathomimetic agonist	Myocardial infarction (Wiener et al., 1990). Cardiac arrhythmias; sudden death (Haller and Benowitz, 2000; Samenuk et al., 2002). Cerebrovascular accident (Bruno et al., 1993).
Phenylpropanolamine	Sympathomimetic agonist	Haemorrhagic/ischemic strokes (Kernan et al., 2000). Myocardial infarction (Leo et al., 1996). Hypertensive crisis (Lake et al., 1990).

## Conclusion

This review identifies significant differences in the development and function of WAT, BAT, and beige adipocytes. While BAT activation increases energy expenditure and improved energy homeostasis may assist in controlling obesity, the negative implications of non-selective activation of the sympathetic nervous system on the cardiovascular system are also clear. The neuro-circuitry of thermoregulation and knowledge of the neurochemical and functional properties of BAT activation are quite well understood in rodents. However, more research is needed into what primary β-adrenoreceptor is involved in human BAT activation, as this remains a contentious topic. This will assist in better understanding the function of BAT in humans and aid in translating findings from animal studies into human models. Whether caffeine evoked BAT thermogenesis in humans is a result of systemic or central activation remains unclear, however it is clear that caffeine evoked BAT thermogenesis in rodents is a result of central activation. Caffeine potentially exerts its effects on human BAT via acting on the neural pathway underlying metabolism. In particular, through activating orexinergic neurons in the perifornical lateral hypothalamus and lateral hypothalamus, and the antagonism of adenosine receptors more broadly.

## Author Contributions

LVS wrote the manuscript with the rest of contributing revisions and input from JAR, RG, CK, and HRI. All authors contributed to scientific review and discussion of the manuscript.

## Conflict of Interest

The authors declare that the research was conducted in the absence of any commercial or financial relationships that could be construed as a potential conflict of interest.
